# Illegitimate Advertising

**Published:** 1878-01

**Authors:** 


Illegitimate Advertising.—Justice to the readers of medical journals requires that the pages devoted to reading matter should not be occupied with articles laudatory of the preparations of particular manufacturers. We are occasionally presented with such articles for the Journal. While we desire to furnish our readers with all that is new and useful in medicine, we do not think the description of articles referred to, afford advantages to physicians, while they work injustice to other manufacturers of the same articles, who pay for their page in our advertising department. Well-meaning, honorable physicians, who have tested preparations sent them for the purpose, are induced by the donor to write approvingly of the particular manufactory from which they were sent. Such communications seem not to be objectionable, but if accepted indiscriminately by journalists, their original department would soon be filled with articles laudatory of particular pharmacists, affording no practical benefit to physicians. We have, therefore, refused articles of this kind from good medical writers proposing to make regular contributors, but who, we have reason to believe, are paid by manufacturing druggists to make the articles so as to serve as useful advertisements. Now, while we think reliable and faithful pharmacists, affording active, efficient preparations, should be encouraged by making them notorious as such, we think a hired laudator is not the proper person for this business. All our advertisers, so far as we know, have an equal claim upon our confidence, and we cannot, with propriety, and with justice to the medical profession and dealers in medicines, advise a preference, nor allow the JOURNAL to be made the medium of such advice from others. Our duty being first and greatest to our medical readers and their suffering patients, we feel bound to expose any fraudulent preparation likely to prove injurious, and also to express our opinion of new remedies and their preparations, or new preparations of old remedies, by whomsoever discovered or invented.

While we probably could not justly say that there should be any preference for the same alkaloid, or its salts, as prepared by different pharmacists, yet the comparative value of various alkaloids, or the several salts of any one, may afford a practical subject for discussion and interchange of opinion. For example, quinine, salicin, etc., are equally reliable from any respectable pharmacist, but the comparative value of quinia, cinchonia and cinchonidia, whether each be the preparation of different manufacturers or not, is a subject upon which all should express their experience and preference, without regard to the originator or vender of the articles.

